# Measuring severe stroke: a scoping review of RCTs

**DOI:** 10.3389/fneur.2025.1631275

**Published:** 2025-07-30

**Authors:** Katrin Roesner, Hanna Brodowski, Nicole Strutz

**Affiliations:** ^1^Department of Physiotherapy, Pain and Exercise Research Luebeck, Institute of Health Sciences, Universität zu Lübeck, Lübeck, Germany; ^2^International Graduate Academy, Institute of Health and Nursing Sciences, Medical Faculty of Martin Luther University Halle-Wittenberg, University Medicine Halle, Halle (Saale), Germany; ^3^Department of Orthopedic and Trauma Surgery, Martin-Luther-University Halle-Wittenberg, Halle (Saale), Germany

**Keywords:** stroke severity, outcome measure, cut-off scores, neurological rehabilitation, stroke phase, NIHSS

## Abstract

**Background:**

Stroke severity affects length of hospital stay and functional recovery in rehabilitation. Therefore, establishing baseline data of stroke severity is a crucial step. In 2017, neurorehabilitation researchers met at the Stroke Recovery and Rehabilitation Roundtable (SRRR) to build a consensus on new standards for stroke recovery research. Core outcomes for measurement in stroke trials resulted in the recommendation that severe stroke should be assessed using the NIHSS. This scoping review aims to provide an overview of the variety of measurements used in clinical research to assess severe stroke.

**Methods:**

RCTs and CCTs were identified by searching PubMed, CENTRAL, SSCI, and ICTRP, covering articles published between January 2018 and September 2024. Peer-reviewed articles in English focusing on rehabilitative interventions and patients aged 18 years or older who have been classified with a severe stroke. The articles included were analyzed according to used measurements and cut-off scores.

**Results:**

The initial search yielded 1,004 publications, of which 35 (3.6%) studies were deemed eligible. In total, 11 different measures were used to assess severe stroke. Most studies used the NIHSS (*n* = 14), followed by mRS (*n* = 6), the FMA upper extremity (*n* = 4), the original FMA (*n* = 4) and the (modified) BI (*n* = 3). Seven different cut-off scores for the NIHSS were identified, with the scale being most frequently used in clinical settings.

**Conclusion:**

This review indicates substantial variability in measurements and a diverse range of cut-off scores. Consequently, comparability of patients’ baseline stroke severity across studies is limited. Given the fact that the NIHSS is only partially used, future efforts should focus on barriers and challenges using the NIHSS.

## 1 Introduction

Strokes affect more than one billion people worldwide and are the leading cause of disability and the second leading cause of death (1). Post-stroke consequences can be reflected at every level of the International Classification of Functioning, Disability and Health (2). Different standardized measurements address these domains, capturing the complex impact of stroke on function, activity, and participation. An important factor influencing stroke survivors’ outcomes is stroke severity (3, 4). It is a key factor in hospital length of stay, which is one of the most important indicators for monitoring the utilization of hospital treatment (5). Unfortunately, the definition of stroke severity, especially severe stroke, is not used uniformly, with a wide range of different measures found (6–8).

In 2017, the measurement working group of the ‘Stroke Recovery and Rehabilitation Roundtable’ (SRRR) was established to develop standardized recommendations and establish guidelines for standardized measuring time points and metrics to be used in all adult stroke sensorimotor recovery research (9). According to the SRRR, the ‘National Institute of Health Stroke Scale’ (NIHSS) should be used as a baseline measurement to determine the severity of a stroke, providing a quantifiable measurement of post-stroke neurological impairments across domains as well as the severity of symptoms linked to cerebral infarcts (9).

The NIHS Scale ranges between 0 and 42 points, with a higher score indicating a higher stroke severity. Based on the work of Brott et al., the most prevalent cut-off values of the NIHSS for defining stroke severity are labeled as mild (1–4), moderate (5–14), severe (15–24) and very severe (25+) (10). Briggs et al. used a cut-off score of >16 points to define a severe stroke as well as >20/42 (11).

It is not known how the severity of stroke is currently classified in clinical research or whether they are measured using the recommended NIHSS. This scoping review aims to provide an overview of stroke severity measurements and cut-off scores used in clinical rehabilitation research.

## 2 Methods

### 2.1 Study design

This scoping review was conducted according to the Joanna Briggs Institute guideline for scoping research and reported in accordance with the Preferred Reporting Items for Systematic Reviews and Meta-Analyses statement for reporting scoping (12). The study protocol was pre-registered on the Open Science Framework Platform.^1^

### 2.2 Information sources and search strategy

Searches were conducted between January 2018 and September 2024 using a specified search string (Table 1; Supplementary material). After an initial search, a comprehensive search strategy was developed and applied to MEDLINE via PubMed, the Cochrane Central Register of Controlled Trials (CENTRAL), the Social Sciences Citation Index (SSCI), and the International Clinical Trials Registry Platform (ICTRP).

**Table 1:** Search string.

MEDLINE via PubMed			
1 severe stroke[Title/Abstract] 2 stroke severity[Title/Abstract] 3 stroke disab*[Title/Abstract] 4 severe stroke impair*[Title/Abstract] 5 severe stroke limit*[Title/Abstract] 6 #1 OR #2 OR #3 OR #4 OR #5 7 Autogenic Training[MeSH Terms] 8 Combined Modality Therapy[MeSH Terms] 9 Exercise Movement Techniques[MeSH Terms] 10 Mentoring[MeSH Terms] 11 Nursing Care[MeSH Terms] 12 Patient Positioning[MeSH Terms] 13 Stroke Rehabilitation[MeSH Terms] 14 Teaching[MeSH Terms] 15 Transcutaneous Electric Nerve Stimulation[MeSH Terms]	16 Video Games[MeSH Terms] 17 Virtual Reality Exposure Therapy[MeSH Terms] 18 aerobic exercise[Title/Abstract] 19 aerobic training[Title/Abstract] 20 biofeedback[Title/Abstract] 21 coaching[Title/Abstract] 22 cognitive behavioral therapy[Title/Abstract] 23 cognitive rehabilitation[Title/Abstract] 24 constraint induced therapy[Title/Abstract] 25 education[Title/Abstract] 26 electric stimulation therapy[Title/Abstract] 27 exercise[Title/Abstract] 28 exercise therapy[Title/Abstract] 29 functional electric stimulation[Title/Abstract] 30 health education[Title/Abstract]	31 home care[Title/Abstract] 32 home rehabilitation[Title/Abstract] 33 intensive care[Title/Abstract] 34 mirror therapy[Title/Abstract] 35 mobilization[Title/Abstract] 36 motor relearning program[Title/Abstract] 37 movement therapy[Title/Abstract] 38 muscle training[Title/Abstract] 39 neuromuscular electric stimulation[Title/Abstract] 40 nursing[Title/Abstract] 41 occupational therapy[Title/Abstract] 42 patient education[Title/Abstract] 43 physical activity[Title/Abstract] 44 physical therapy modalities[Title/Abstract] 45 physical therapy speciality[Title/Abstract]	46 robotics[Title/Abstract] 47 task specific training[Title/Abstract] 48 virtual reality[Title/Abstract] 49 #7 OR #8 OR #9 OR #10 OR #11 OR #12 OR #13 OR #14 OR #15 OR #16 OR #17 OR #18 OR #19 OR #20 OR #21 OR #22 OR #23 OR #24 OR #25 OR #26 OR #27 OR #28 OR #29 OR #30 OR #31 OR #32 OR #33 OR #34 OR #35 OR #36 OR #37 OR #38 OR #39 OR #40 OR #41 OR #42 OR #43 OR #44 OR #45 OR #46 OR #47 OR #48 50 #6 AND #49 51 #50 (Filter: Randomized Controlled Trial) 52 #51 (Filter: Date - Publication 01.01.2018 to 06.09)

### 2.3 Inclusion/exclusion criteria

All RCTs and CCTs published in peer-reviewed journals enrolled severe stroke patients aged ≥18 years undergoing a rehabilitative intervention (e.g., physiotherapy, speech-language therapy, occupational therapy, nursing, and neuropsychology) were included. Articles were excluded if they used pharmacological and surgical interventions, non-invasive brain stimulation and complementary or alternative medicine interventions. If one or more secondary analyses were published, it was checked whether the primary study had already been included, if not, the first publication of a secondary analysis was included.

### 2.4 Study selection

The Rayyan management software (Rayyan Systems Inc., Cambridge, MA 02142, United States) was used to select the included articles (13). Two reviewers (KR, LK) independently screened titles and abstracts to exclude those that did not meet the inclusion criteria. In a second step, the same two researchers independently performed a full-text screening of the remaining studies etiology. Disagreements during the entire process were discussed with a third researcher (NS) until consensus was reached.

### 2.5 Data extraction

The data from each included study was extracted by KR and HB, using a data extraction framework. Conflicts were resolved by discussion with NS. According to the definition of scoping reviews, the methodological quality of the included studies was not evaluated. Following the JBI methodology for scoping reviews, a formal appraisal of methodological quality was not required (14).

Stroke severity measurement tools used in the included studies were grouped according to the International Classification of Functioning, Disability and Health into body function and body structures, activities, and participation (15).

## 3 Results

**Figure 1:**
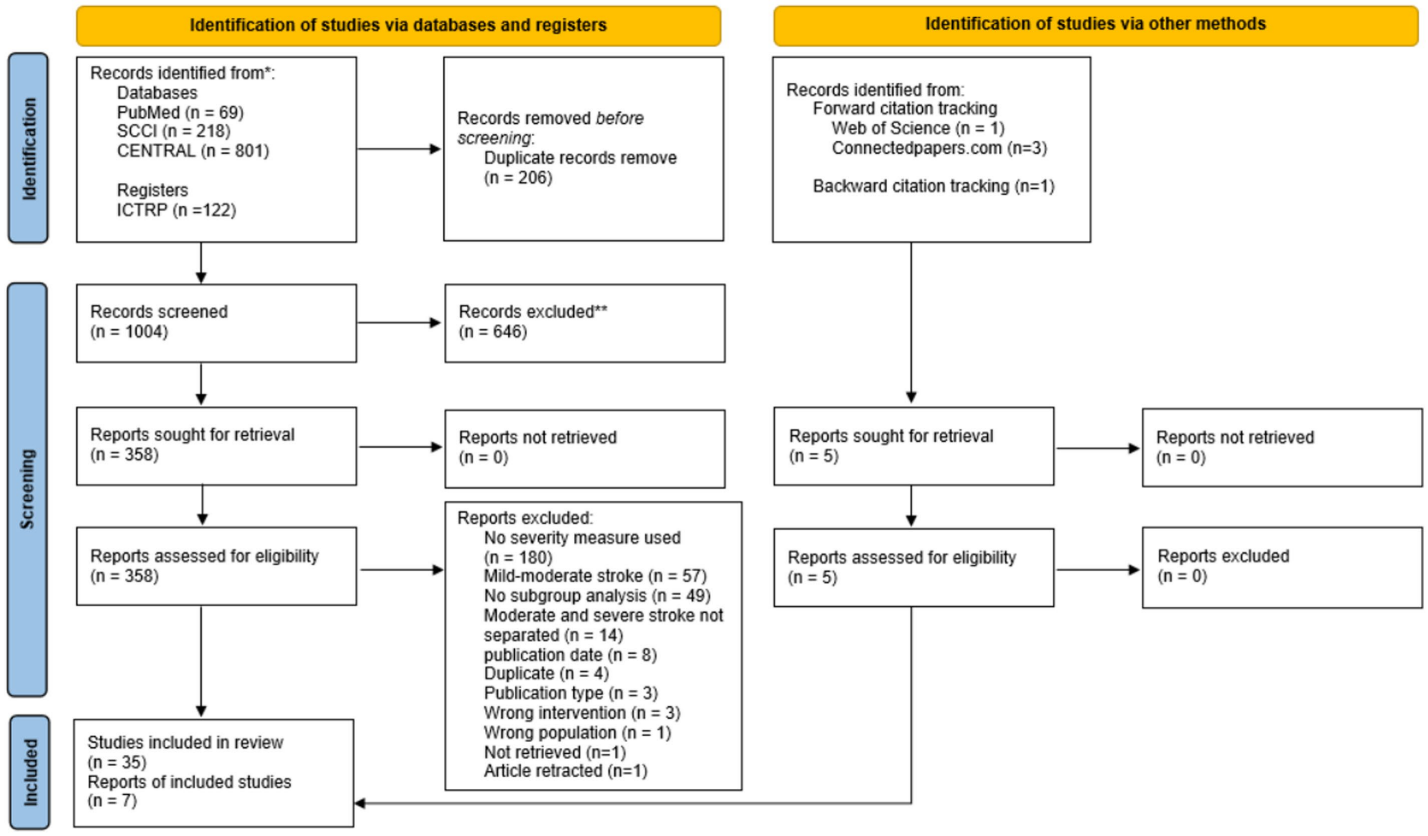
Study selection flow chart according to PRISMA 2020.

In total, 1,004 articles were identified for screening. After screening titles and abstracts, 646 articles were excluded due to exclusion criteria like pharmacological therapy or congress contribution without conclusive results. A total of 358 references remained and were screened for inclusion. The complete process for the inclusion of the final 35 publications is depicted in the PRISMA flow chart (Figure 1).

### 3.1 Description of included studies

Out of 1,004 articles screened, 35 articles were included. Geographically, most of the studies were conducted in Europe (38%), followed by America (24%), Asia (24%), and Australia (14%). Participants were recruited in various settings, which were categorized into three groups. Starting with the clinical setting (*n* = 15) and the rehabilitation setting (*n* = 14). The term “non-clinical/rehabilitative setting” (*n* = 10) was used to categorize various settings—such as laboratory or community settings—that did not align with either of the two primary categories. In two studies (16, 17) participants were recruited in two settings. In the studies by Mulder et al. (18) and Sakakibara et al. (19), participants were recruited from three different settings. Salazar et al. (20) did not provide any information about the setting.

Within these studies, participants were mostly included during the early subacute phase (*n* = 13), followed by chronic stroke phase (*n* = 8), hyperacute stroke phase (*n* = 8), late subacute (*n* = 3), and acute (*n* = 2). For two of these studies, the stroke phase was not specified.

Most interventions involved physical activation, including specific exercises, training programs, or early mobilization. Additionally, many interventions incorporated robotic-assisted technologies or other digital health solutions, such as mobile applications, health platforms, or virtual reality. Several studies implemented transcranial stimulation and brain-computer interfaces as part of the intervention. A smaller proportion received video-based education or adherence-enhancing strategies. Most of the control group received conventional therapy, standard hospital care, and home exercise program for the clinic or rehabilitation facility (Table 2).

**Table 2:** Included studies, settings, stroke phases, stroke severity measurements and interventions of RCTs and CCTs.

Author, date, location	*n*	Setting	Phase	Measurement	Intervention
Aguiar et al. (2020) (25) Brazil, Canada	22	Non clinical/rehab	Chronic	FMA-UL FMA-LL	IG: aerobic treadmill training CG: outdoor-overground walking
Aprile et al. (2020) (47) Italy	247	Rehab	Early and late subacute	FMA-UL	IG: robotic and sensor-based device CG: conventional treatment:
Brunner et al. (2024) (29) Denmark	40	Rehab	Early subacute	NIHSS	IG: sessions with Brain-Computer Interfaces CG: standard physiotherapy and occupational therapy
Buvarp et al. (2023) (42) Sweden	1.367	Clinical, rehab	Hyperacute, acute	NIHSS	IG: increased physical activity CG: decreased physical activity
Chen et al. (2021) (27) China	96	Non clinical/rehab	Acute	BI	IG: goal-oriented Intervention CG: health education
Conroy et al. (2019) (24) United States	45	Clinical	Chronic	FMA	IG: robot-assisted arm training CG: therapist-assisted task training
Cumming et al. (2018, 2019) (48, 49), Walters et al. (2020) (50), Bernhardt et al. (2021) (51) United Kingdom, Australia, South East Asia	2.104	Clinical	Hyperacute	NIHSS	IG: very early and more frequent mobilization CG: usual care
De Bruyn et al. (2020) (30) Belgium	40	Rehab	Early subacute	FMA	IG: sensorimotor therapy (SENSe approach) and task specific exercises CG: cognitive table-top games with the non-affected UL and 30 min task-specific motor exercises
De Jong et al. (2018) (52) United Kingdom, Netherlands	46		Early subacute	FMA-UL	IG: PT therapy as recommended by the Dutch stroke guideline CG: OT therapy as recommended by the Dutch stroke guideline
Ertas-Spantgar et al. (2024) (28) Germany	24	Rehab	Post-acute	BI	IG: usual therapy and experimental therapy by RehaGoal App CG: usual therapy
Frange et al. (2023) (21) Brazil	8	Clinical	Hyperacute	NIHSS	IG: performing calf muscle contractions by activity CG: inactivity
Kamal et al. (2020) (43) Pakistan	310	Clinical	Acute	NIHSS	IG: video-based education intervention CG: standard care
Kersey et al. (2023) (16) United States	32	CLINICAL and non clinical/rehab	Hyperacute, acute, early subacute	NIHSS	IG: patients receive strategy training using a mobile health platform (iADAPT) CG: patients receive strategy training using a workbook
Kim et al. (2020) (53) Korea	21	Rehab	Late subacute	FAC	IG: underwater gait training CG: overground gait training
Koo et al. (2018) (31) Korea	24	Clinical	Early subacute	mBI	IG: anodal transcranial direct current stimulation CG: sham stimulation
Logan et al. (2022) (54) England	45	Clinical	Hyperacute, acute	mRS	IG: functional standing frame program CG: usual physiotherapy
Le Franc et al. (2021) (55) France	20	Rehab	Chronic	FMA-UL	IG: tendor vibration and visual feedback; severe stroke group CG: tendor vibration and visual feedback; mild to moderate stroke group
Mahmood et al. (2022) (17) India		Rehab and non-clinical/rehab	Early subacute, chronic	FMA	IG: received additional adherence strategies CG: standard hospital care and home exercise program
Martins et al. (2020) (32) Brazil, Canada	26	Nonclinical/rehab	Chronic	FMA-UL FMA-LL	IG: task-specific circuit training CG: standard care
Middleton et al. (2019) (56) Australia	970	Clinical	Hyperacute to acute	LAMS	IG: Intervention stroke units with treatment protocols CG: Control group stroke units
Mulder et al. (2022) (18) Australia, Netherlands	129	Clinical, rehab and non-clinical/rehab	Early and late subacute	mRS	IG: 8-week caregiver-mediated exercises intervention CG: control intervention
Nagai et al. (2024) (57) Japan	42	Clinical	n.n.	mRS FIM motor item	IG: standing on unstable board for the nonparalyzed lower limbs CG: usual physical therapy
Ouyang et al. (2020, 2021) (44, 58) China, United Kingdom, Australia, India, Sri Lanka	11.084	Clinical	Hyperacute	NIHSS	IG: sitting up head position CG: lying flat
Radford et al. (2020) (22) n.n.	46	Clinical	n.n.	NIHSS	IG: vocational rehabilitation CG: usual care
Renner et al. (2020) (59) Germany	69	Rehab	Late subacute	FMA-UL MRC	IG: bilateral training CG: unilateral training
Reynolds et al. (2021) (33) Australia	20	Rehab	Early subacute	NIHSS mRS	IG: moderate-intensity fitness training CG: low-intensity exercise
Rose et al. (2022) (34) Australia, New Zealand	116	Non-clinical/rehab	Chronic	mRS	IG: Aphasia Therapies CIAT-Plus or M-MAT CG: usual care
Sakakibara et al. (2022) (19) Canada	126	Clinical, rehab and Non-clinical/rehab	Chronic	mRS	IG: Stroke Coach CG: Memory Training group
Salazar et al. (2024) (20) United States	20	n. n.	Chronic	NIHSS	IG: transcranial direct current stimulation CG: sham during image acquisition
Smith et al. (2021) (45) United States	23	Rehab	Early subacute	NIHSS	IG: bimanual lever-driven wheelchair CG: Conventional exercise group
Stockbridge et al. (2023) (35) United States	51	Clinical	Chronic	NIHSS	IG: transcranial direct current stimulation (tDCS) CG: standard care
Straudi et al. (2020) (26) Italy	39	Rehab	Early subacute	FMA-LL	IG: robot-assisted arm therapy and hand functional electrical stimulation CG: time-matched intensive conventional therapy
Threapleton et al. (2020) (23) United Kingdom	33	Clinical	All phases	NIHSS OSCPS	IG: virtual reality intervention CG: usual care
Tistad et al. (2018) (36) Sweden	237	Rehab	Early subacute	BI	IG: client-centered activities of daily living intervention CG: usual activities of daily living intervention
Watkins et al. (2022) (37) United Kingdom	157	Clinical	Hyperacute	NIHSS	IG: systematic voiding program for urinary incontinence CG: usual care

BI, Barthel Index; CG, control group; FAC, Functional Ambulation Categories; FMA, Fugl-Meyer-Assessment; FMA-UL, Fugl-Meyer-Assessment upper extremity; FMA LL, Fugl-Meyer-Assessment lower extremity; IG, intervention group; LAMS, Los Angeles Motor Scale; mBI, Modified Barthel Index; mRS, modified Rankin Scale; NIHSS, National Institute Stroke Scale; OCSPC, Oxfordshire Community Stroke Project Classification.

### 3.2 Identified measures and framework conditions

Eleven different measures were used to assess stroke severity (Table 3). Most studies used the NIHSS (*n* = 14), followed by modified Rankin Scale (*n* = 6). The Fugl-Meyer-Assessment for the upper extremity (FMA-UL), and the original Fugl-Meyer-Assessment (FMA), were each used four times to address stroke severity. The Barthel Index (BI) was used in three studies, and the Functional Ambulation Categories (FAC) and the Los Angeles Motor Scale (LAMS) was used in one study. Six studies included two measures to assess stroke severity. The NIHSS was used in seven studies to identify the hyperacute phase.

**Table 3:** Overview Stroke Measurements and ICF categories; *n*=11

**Instrument of Measurement (abbrev.)**	**Original authors**	**Method of report**	**Components**	**Scoring system**	**Validation studies**
National Institute of Health Stroke Scale (NIHSS)	Brott, T., Adams, H. P., Jr, Olinger, C. P., Marler, J. R., Barsan, W. G., Biller, J., Spilker, J., Holleran, R., Eberle, R., & Hertzberg, V. (1989). Measurements of acute cerebral infarction: a clinical examination scale. Stroke, 20(7), 864–870. https://doi.org/10.1161/01.str.20.7.864	Observer Paper and Pencil	15 Items Aphasia, Behavior, Cognition, Dysarthria, Vision Perception	0 - 3 and 0 - 4 ordinal scale with written and numerical descriptors. Calculation of a total score 0 - 42. Higher scores indicate greater severity.	Brott T, Adams HP Jr, Olinger CP, Marler JR, Barsan WG, Biller J, Spilker J, Holleran R, Eberle R, Hertzberg V, et al. Measurements of acute cerebral infarction: a clinical examination scale. Stroke. 1989 Jul;20(7):864-70. doi: 10.1161/01.str.20.7.864. PMID: 2749846.
Fugl-Meyer-Assessment (FMA)	Fugl-Meyer, A. R., Jääskö, L., Leyman, I., Olsson, S., & Steglind, S. (1975). The post-stroke hemiplegic patient. 1. a method for evaluation of physical performance. *Scandinavian journal of rehabilitation medicine*, *7*(1), 13–31.	Observer Paper & Pencil	155 Items Activities of daily linving, Functional Mobility, Pain Five domains (Motor function, Sensory function, Balance, Joint range of Motion, Joint pain),	0 - 3 ordinal scale Calculation of a total score 0 – 226	Fugl-Meyer AR et al.: The post-stroke hemiplegic patient. A method for evaluation of physical performance. Scand J Rehabil Med 1975, 7:13-31
Fugl-Meyer upper extremity (FM UE) (as part of the FMA)	Fugl-Meyer, A. R., Jääskö, L., Leyman, I., Olsson, S., & Steglind, S. (1975). The post-stroke hemiplegic patient. 1. a method for evaluation of physical performance. *Scandinavian journal of rehabilitation medicine*, *7*(1), 13–31.	Observer Paper & Pencil	A - Shoulder/Elbow /Forearm B – Wrist C – Hand D – Coordination/ Speed	0 - 3 ordinal scale Calculation of a total score 0 - 66	Fugl-Meyer AR et al.: The post-stroke hemiplegic patient. A method for evaluation of physical performance. Scand J Rehabil Med 1975, 7:13-31
Fugl-Meyer lower extremity (FM LE) (as part of the FMA)	Fugl-Meyer, A. R., Jääskö, L., Leyman, I., Olsson, S., & Steglind, S. (1975). The post-stroke hemiplegic patient. 1. a method for evaluation of physical performance. *Scandinavian journal of rehabilitation medicine*, *7*(1), 13–31.	Observer Paper & Pencil	E – Hip/Knee/Ancle F – Coordination/ Speed G - Balance	0 - 3 ordinal scale Calculation of a total score 0 - 34	Fugl-Meyer AR et al.: The post-stroke hemiplegic patient. A method for evaluation of physical performance. Scand J Rehabil Med 1975, 7:13-31
Barthel Index (BI)	Mahoney, F. I., & Barthel, D. W. (1965). Functional evaluation: the barthel index. *Maryland state medical journal*, *14*, 61–65.	Performance Measure Paper & Pencil	10 Items Activities of Daily Living Functional Mobility Gait	Items are rated based on the amount of assistance required to complete each activity, can choose between 0-5-10-15	Collin C, Wade DT, Davies S, Horne V. The Barthel ADL Index: a reliability study. Int Disabil Stud. 1988;10(2):61-3. doi: 10.3109/09638288809164103. PMID: 3403500.
Modified Barthel Index (mBI)	Collin, C., Wade, D. T., Davies, S., & Horne, V. (1988). The Barthel ADL Index: a reliability study. *International disability studies*, *10*(2), 61–63. https://doi.org/10.3109/09638288809164103	Performance Measure Paper & Pencil	10 Items Activities of Daily Living Functional Mobility Gait	Items are rated based on the amount of assistance required to complete each activity	Shah, S, Vanley, F., Cooper, B. Improving the sensitivity of the Barthel Index for stroke rehabilitation https://doi.org/10.1016/0895-4356(89)90065-6
Modified Rankin Scale (mRS)	van Swieten, J. C., Koudstaal, P. J., Visser, M. C., Schouten, H. J., & van Gijn, J. (1988). Interobserver agreement for the assessment of handicap in stroke patients. *Stroke*, *19*(5), 604–607. https://doi.org/10.1161/01.str.19.5.604	Observer	Single item	6-point rankin scale fom 0=no symptoms to 5=severe disability: bedridden, incontinent, and requiring constant nursing care and attention	Rankin, J. (1957). Cerebral vascular accidents in patients over the age of 60. *Scott Med J*, 2, 200-215.
Functional Ambulation Categories (FAC)	Holden M.K., Gill K.M., Magliozzi M.R., Nathan J., Piehl-Baker L. Clinical gait assessment in the neurologically impaired Reliability and meaningfulness. *Phys Ther.* 1984; 64: 35-40	Observer Paper & Pencil	Assessing walking ability in 6 broard categories	0-5 Higher score indicates less dependency	Mehrholz, J., Wagner, K., Rutte, K., Meiner, D. and Pohl, M. Predictive validity and responsiveness of the Functional Ambulation Category in hemiparetic patients after stroke. Archives of Physical Medicine Rehabilitation, 2007, 88, 1314-1319.
Action Research Arm Test	Lyle R. C. (1981). A performance test for assessment of upper limb function in physical rehabilitation treatment and research. *International journal of rehabilitation research. Internationale Zeitschrift fur Rehabilitationsforschung. Revue internationale de recherches de readaptation*, *4*(4), 483–492. https://doi.org/10.1097/00004356-198112000-00001	Observer Paper & Pencil	Activities of Daily Living, Coordination, Dexterity, Upper Extremity Function 19 Items	4-point ordinal scale Calculation of a total score 0 - 57	Koh CL, Hsueh IP, Wang WC, Sheu CF, Yu TY, Wang CH, Hsieh CL. Validation of the action research arm test using item response theory in patients after stroke. J Rehabil Med. 2006 Nov;38(6):375-80. doi: 10.1080/16501970600803252. PMID: 17067971. Chen HF, Lin KC, Wu CY, Chen CL. Rasch validation and predictive validity of the action research arm test in patients receiving stroke rehabilitation. Arch Phys Med Rehabil. 2012 Jun;93(6):1039-45. doi: 10.1016/j.apmr.2011.11.033. Epub 2012 Mar 14. PMID: 22420887.
Oxfordshire Community Stroke Project Classification (OCSP)	Bamford, J., Sandercock, P., Dennis, M., Burn, J., & Warlow, C. (1991). Classification and natural history of clinically identifiable subtypes of cerebral infarction. *Lancet (London, England)*, *337*(8756), 1521–1526. https://doi.org/10.1016/0140-6736(91)93206-o		classification of four sub-categories of cerebral infarction based on presenting symptoms and signs:	lacunar infarcts (LACI) total anterior circulation infarcts (TACI) partial anterior circulation infarcts (PACI) posterior circulation infarcts (POCI)	A prospective study of acute cerebrovascular disease in the community: the Oxfordshire Community Stroke Project 1981-86. 1. Methodology, demography and incident cases of first-ever stroke.J Neurol Neurosurg Psychiatry. 1988 Nov; 51(11): 1373–1380.
Los Angeles Motor Scale (LAMS)	Llanes, J. N., Kidwell, C. S., Starkman, S., Leary, M. C., Eckstein, M., & Saver, J. L. (2004). The Los Angeles Motor Scale (LAMS): a new measure to characterize stroke severity in the field. *Prehospital emergency care*, *8*(1), 46–50. https://doi.org/10.1080/312703002806	Observer	3-item prehospital scoring tool Facial droop Arm drift Grip strength	0-1 0=absent +1= present/drifts down, weak grip +2=falls rapidly/no grip	Kim JT, Chung PW, Starkman S, et al. Field Validation of the Los Angeles Motor Scale as a Tool for Paramedic Assessment of Stroke Severity. Stroke. 2017; 48(2): 298-306. Nazliel B., Starkman S, Liebeskind DS, et al. A brief prehospital stroke severity scale identifies ischemic stroke patients harboring perstisting large arteroaö occlusions. Stroke. 2008; 39(8): 2264-7.

A, Activity; BF, Body function; I, Impairment; ICF, International Classification of Functioning, Disability and Health; P, Participation.

The original FMA was used in all three settings, mainly during the chronic phase, as well as the combination of FMA-UL and the Fugl-Meyer-Assessment of the lower extremity (FMA-LL) (n = 3) and once during the early subacute phase. In contrast, the FMA-UL was used twice in a rehabilitation setting during early subacute and late subacute.

Most of the measures can be assigned to a single ICF level. The NIHSS, Oxfordshire Community Stroke Project Classification (OCSP), and Los Angeles Motor Scale (LAMS) correspond to body function and impairment. Only the FMA as well as FMA-UL and FMA-LL, and the Functional Independence Measure (FIM) motor items cover both body function and impairment as well as the activity level. The remaining five measures are classified under the activity level.

**Figure 2:**
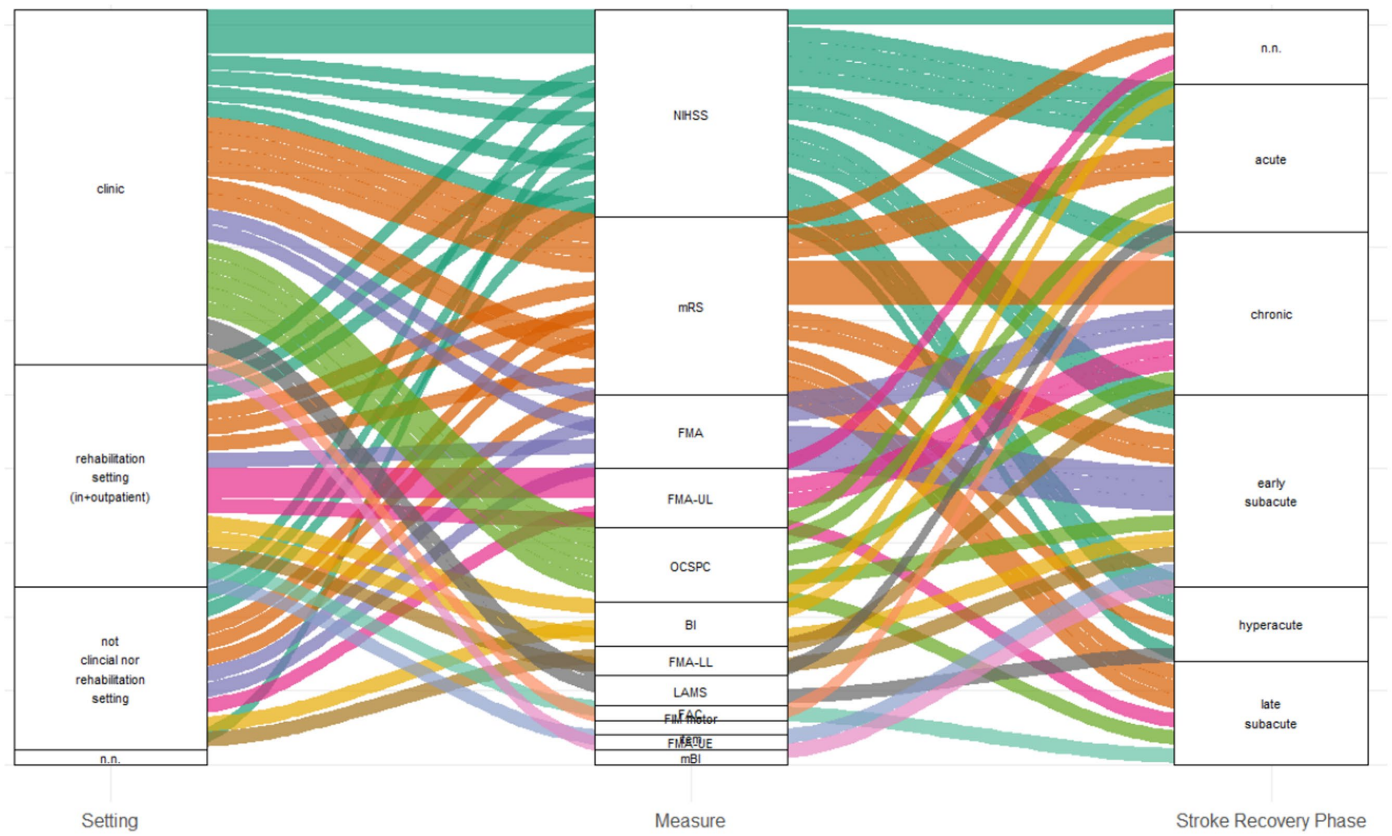
Sankey diagram of setting, measurement, and phase. BI, Barthel Index; CG, control group; FAC, Functional Ambulation Categories; FMA, Fugl-Meyer-Assessment; FMA-UL, Fugl-Meyer-Assessment upper extremity; FMA LL, Fugl-Meyer-Assessment lower extremity; IG, intervention group; LAMS, Los Angeles Motor Scale; mBI, Modified Barthel Index; mRS, modified Rankin Scale; NIHSS, National Institute Stroke Scale; OCSPC, Oxfordshire Community Stroke Project Classification.

Figure 2 illustrates the relationships among the three domains—“setting,” “measurement,” and “stroke recovery phase”—using connections of varying widths, where the thickness of each connection reflects the number of shared elements between the domains.

### 3.3 Cut-off scores

**Figure 3:**
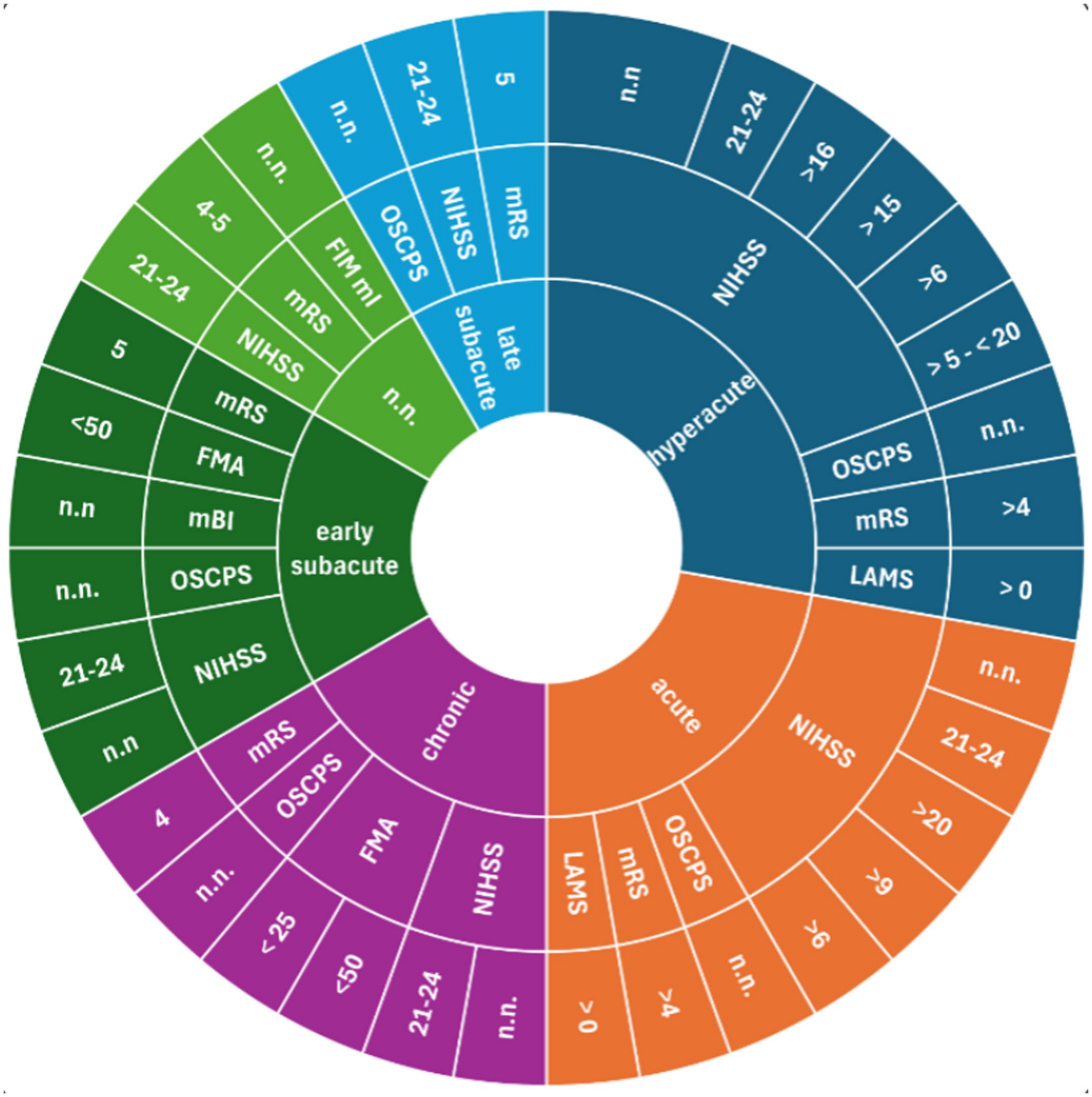
Measurements assessing severe stroke used in clinical settings; inner ring: stroke recovery phase; middle ring: measurements; outer ring: cut-off scores. BI, Barthel Index; FAC, Functional Ambulation Categories; FMA, Fugl-Meyer-Assessment; FMA LL, Fugl-Meyer-Assessments lower limb; FMA-UL, Fugl-Meyer-Assessment upper limb; LAMS, Los Angeles Motor Scale; mBI, modified Barthel Index; mRS, modified Rankin Scale; NIHSS, National Institute Stroke Scale; n.n., not named.

**Figure 4:**
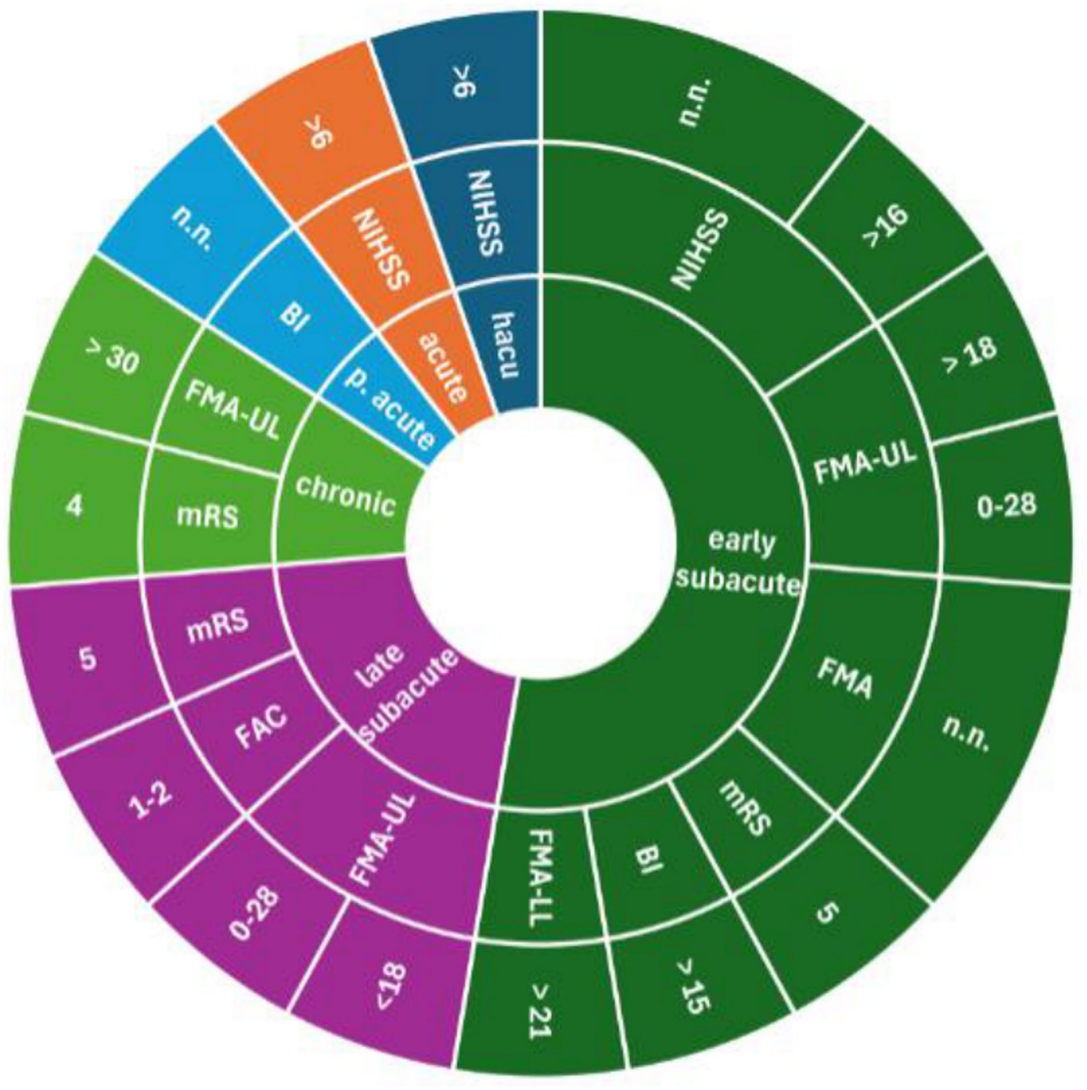
Measurements assessing severe stroke used in rehabilitation settings; inner ring: stroke recovery phase; middle ring: measurements; outer ring: cut-off scores. BI, Barthel Index; FAC, Functional Ambulation Categories; FMA, Fugl-Meyer-Assessment; FMA LL, Fugl-Meyer-Assessments lower limb; FMA-UL, Fugl-Meyer-Assessment upper limb; LAMS, Los Angeles Motor Scale; mBI, modified Barthel Index; mRS, modified Rankin Scale; NIHSS, National Institute Stroke Scale; n.n., not named.

**Figure 5:**
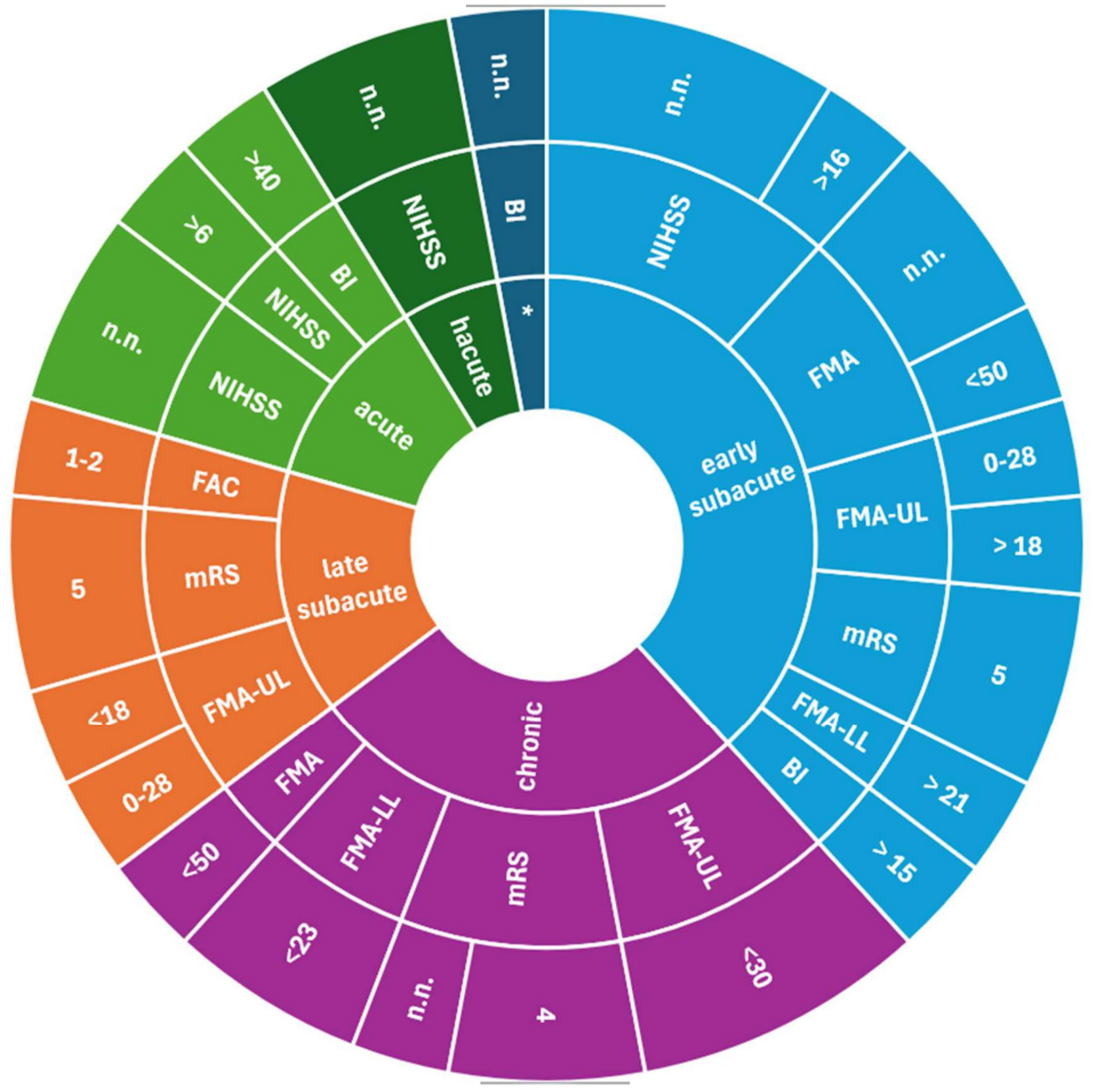
Measurements assessing severe stroke used in not clinical/rehabilitation settings; inner ring: stroke recovery phase; middle ring: measurements; outer ring: cut-off scores. BI, Barthel Index; FAC, Functional Ambulation Categories; FMA, Fugl-Meyer-Assessment; FMA LL, Fugl-Meyer-Assessments lower limb; FMA-UL, Fugl-Meyer-Assessment upper limb; LAMS, Los Angeles Motor Scale; mBI, modified Barthel Index; mRS, modified Rankin Scale; NIHSS, National Institute Stroke Scale; n.n., not named.

Study protocols provided different cut-off scores to assess severe stroke (Figures 3–5). For the NIHSS, the range for severe stroke was >5 and <20 (21) to 21–24 (22, 23). For the FMA the cut-off score applied was < 25 (24) and <50 (17). For the FMA-UL, the cut-off score was <30 (25) or ≤ 21 (26). For the BI, a cut-off score of ≤ 40 (27) or <30 (28) was used to assess severe stroke. In 11 studies (16, 20, 29–37), the cut-off scores for the evaluation of the severity of stroke were not specified.

## 4 Discussion

Clinical manifestation of stroke varies depending on factors like etiology, localization, and stroke severity, with initial stroke severity known to be a crucial predictor of outcomes (38). Scales and measurements help to quantify the severity of stroke symptoms, aiding in treatment decisions. The focus of this scoping review was to give an overview of the measurements, and the cut-off scores used in clinical research to classify stroke severity.

Clinical symptoms undergo considerable changes over time. Guidelines consider these diverse areas of post-stroke disability and their associated symptoms beyond the acute phase of the disease. Stroke recovery includes the examination of level of consciousness, overall neurological impairment, motor function, balance, cognition, speech and language, Activities of Daily Living (ADL), depression, family functioning, and quality of life (39). For this study, research protocols focus on different outcomes, which require different methods and measures suitable for the individual research aim. On the one hand, the multitude of measures presented in this current review is not surprising; instead, they reflect the many different post-stroke symptoms and aims of stroke research. On the other hand, various measures to assess stroke lead to limitations in stroke research as the non-uniform use limits the ability to assemble treatment evidence across trials (40, 41). This review counted 11 measurements, underlining the lack of standardization. According to the roundtable, other outcome measures aligned with the trial’s purpose and target intervention can be added. The recommendation applies across all stages, from the hyperacute to the early and late subacute to the chronic phase. For the studies included in this review, it can be stated that this recommendation was not followed in 21 out of 35 studies.

With that in mind, it must be additionally mentioned that completely different constructs are assessed when using the FAC, the FMA-UL or the ARAT, for example. It should be critically questioned whether it is sufficient to determine the severity of a stroke solely based on walking or upper limb function. For the sake of completeness, it must also be said that the NIHSS does not provide information on activities of daily living like walking or transfers, which are crucial factors for patients’ independence and thus for discharge. The results of this scoping review seem to reflect the lack of a single measure capturing all ICF levels. The second part of the current research question referred to quantifying severe stroke. Results showed that the cut-off scores used for identical measurements varied in the included studies. This is especially notable for the NIHSS and the FMA. Among the studies that reported cut-offs for the NIHSS, seven different ones were found. Buvarp et al. (42) indicated a cut-off for severe stroke at >6 points. Kamal et al. (43) at >9 points, Ouyang et al. (44) at >15, Smith et al. (45) at >16, Liu et al. (46) at >20, Frange (21) >5 and >20 and Radford et al. (22) between 21 and 24. Results with a value of 9 out of 42 points can hardly be comparable with one of 20. Similarly, different cut-off values can be found in the results of the FMA with cut-offs less than 25 or less than 50. The authors of the studies included refer to various sources. Without the authors giving more detailed reasons for the cut-off scores used, it can be assumed that the scores are adapted to the respective population and setting. A cut-off score could be comprehensible and appropriate for individual study, but it must be considered, as it limits quantitative synthesis.

### 4.1 Strengths and limitations

This is the first study about the realization of stroke measures focusing on assessing severe stroke and the used cut-off scores. One strength of this review is the comprehensive search strategy specific to non-medical therapeutic interventions in stroke rehabilitation. Furthermore, the research team provides a diverse educational/professional background in treating severe stroke patients.

A reason for the limited number of search results was the inclusion of CCT and RCT study types. Because there is extensive research in the neurorehabilitation of stroke, the quality of these studies also provided the opportunity to include studies that may be of interest for guideline recommendations.

This review’s wide range of measures reflects the diversity of existing tools for assessing stroke severity. These results highlight the variety of measures used in research and those used in clinical practice to evaluate severe stroke.

## 5 Conclusion

Using different instruments and cut-off scores to assess stroke severity, the measurements’ informative value is limited. It remains unclear what functional abilities the affected person has, as the measurements are based on non-standardized constructs. The categorization and standardization of stroke severity could facilitate communication between healthcare professionals, health insurance companies, and healthcare institutions. This is the case if there is a mutual understanding of stroke severity across all sectors. The use of the NIHSS as a basic instrument, as recommended by the Roundtable, and an instrument addressing the ICF level could reflect the actual situation of patients. Further research is required into obligatory, cross-setting cut-off scores.

## Footnotes

^1^OSF, register number: 10.17605/OSF.IO/WYR5H, https://osf.io/wyr5h
