# Supplementary material for: Measuring severe stroke: a scoping review of RCTs
Source: Front Neurol. 2025 Jul 30;16:1631275. doi: 10.3389/fneur.2025.1631275 (PMC12344423; doi:10.3389/fneur.2025.1631275)
Supplement: Supplementary file 1 [file Table_1.docx]

Supplementary Material

Supplemenray material 1: complete search string

| MEDLINE via PubMed | | | |
| --- | --- | --- | --- |
| 1 severe stroke[Title/Abstract]  2 stroke severity[Title/Abstract]  3 stroke disab*[Title/Abstract]  4 severe stroke impair*[Title/Abstract]  5 severe stroke limit*[Title/Abstract]  6 #1 OR #2 OR #3 OR #4 OR #5  7 Autogenic Training[MeSH Terms]  8 Combined Modality Therapy[MeSH Terms]  9 Exercise Movement Techniques[MeSH Terms]  10 Mentoring[MeSH Terms]  11 Nursing Care[MeSH Terms]  12 Patient Positioning[MeSH Terms]  13 Stroke Rehabilitation[MeSH Terms]  14 Teaching[MeSH Terms]  15 Transcutaneous Electric Nerve Stimulation[MeSH Terms] | 16 Video Games[MeSH Terms]  17 Virtual Reality Exposure Therapy[MeSH Terms]  18 aerobic exercise[Title/Abstract]  19 aerobic training[Title/Abstract]  20 biofeedback[Title/Abstract]  21 coaching[Title/Abstract]  22 cognitive behavioral therapy[Title/Abstract]  23 cognitive rehabilitation[Title/Abstract]  24 constraint induced therapy[Title/Abstract]  25 education[Title/Abstract]  26 electric stimulation therapy[Title/Abstract]  27 exercise[Title/Abstract]  28 exercise therapy[Title/Abstract]  29 functional electric stimulation[Title/Abstract]  30 health education[Title/Abstract] | 31 home care[Title/Abstract]  32 home rehabilitation[Title/Abstract]  33 intensive care[Title/Abstract]  34 mirror therapy[Title/Abstract]  35 mobilisation[Title/Abstract]  36 motor relearning program[Title/Abstract]  37 movement therapy[Title/Abstract]  38 muscle training[Title/Abstract]  39 neuromuscular electric stimulation[Title/Abstract]  40 nursing[Title/Abstract]  41 occupational therapy[Title/Abstract]  42 patient education[Title/Abstract]  43 physical activity[Title/Abstract]  44 physical therapy modalities[Title/Abstract]  45 physical therapy speciality[Title/Abstract] | 46 robotics[Title/Abstract]  47 task specific training[Title/Abstract]  48 virtual reality[Title/Abstract]  49 #7 OR #8 OR #9 OR #10 OR #11 OR #12 OR #13 OR #14 OR #15 OR #16 OR #17  OR #18 OR #19 OR #20 OR #21 OR #22 OR #23 OR #24 OR #25 OR #26 OR #27  OR #28 OR #29 OR #30 OR #31 OR #32 OR #33 OR #34 OR #35 OR #36 OR #37  OR #38 OR #39 OR #40 OR #41 OR #42 OR #43 OR #44 OR #45 OR #46 OR #47  OR #48  50 #6 AND #49  51 #50 (Filter: Randomized Controlled Trial)  52 #51 (Filter: Date - Publication 01.01.2018 to 06.09) |
| *Social Science Citation Index der Web of Science Core Collection* | | | |
| 1 TS=(severe stroke)  2 TS=(stroke severity)  3 TS=(stroke disab*)  4 TS=(severe stroke impair*)  5 TS=(severe stroke limit*)  6 #1 OR #2 OR #3 OR #4 OR #5  7 TS=(autogenic training)  8 TS=(combined modality therapy)  9 TS=(exercise movement techniques)  10 TS=(mentoring)  11 TS=(nursing care)  12 TS=(patient positioning)  13 TS=(stroke rehabilitation)  14 TS=(teaching)  15 TS=(transcutaneous electric nerve stimulation) | 16 TS=(video games)  17 TS=(virtual reality exposure therapy)  18 TS=(aerobic exercise)  19 TS=(aerobic training)  20 TS=(biofeedback)  21 TS=(coaching)  22 TS=(cognitive behavioral therapy)  23 TS=(cognitive rehabilitation)  24 TS=(constraint induced therapy)  25 TS=(education)  26 TS=(electric stimulation therapy)  27 TS=(exercise)  28 TS=(exercise therapy)  29 TS=(functional electric stimulation)  30 TS=(health education) | 31 TS=(home care)  32 TS=(home rehabilitation)  33 TS=(intensive care)  34 TS=(mirror therapy)  35 TS=(mobilisation)  36 TS=(motor relearning program)  37 TS=(movement therapy)  38 TS=(muscle training)  39 TS=(neuromuscular electric stimulation)  40 TS=(nursing)  41 TS=(occupational therapy)  42 TS=(patient education)  43 TS=(physical activity)  44 TS=(physical therapy modalities)  45 TS=(physical therapy speciality) | 46 TS=(robotics)  47 TS=(task specific training)  48 TS=(virtual reality)  49 #7 OR #8 OR #9 OR #10 OR #11 OR #12 OR #13 OR #14 OR #15 OR #16 OR #17 OR  #18 OR #19 OR #20 OR #21 OR #22 OR #23 OR #24 OR #25 OR #26 OR #27 OR #28  OR #29 OR #30 OR #31 OR #32 OR #33 OR #34 OR #35 OR #36 OR #37 OR #38 OR  #39 OR #40 OR #41 OR #42 OR #43 OR #44 OR #45 OR #46 OR #47 OR #48  50 TS=(randomized controlled trial)  51 TS=(RCT)  52 #50 OR #51  53 #6 AND #49 AND #52  54 #6 AND #49 AND #52, Filter: Articles  55 #6 AND #49 AND #52, Filter: Publication Years 2018-2025 |
| *Cochrane Central Register of Controlled Trials* | | | |
| 1 (severe stroke):ti,ab,kw¹  2 (stroke severity):ti,ab,kw  3 (stroke disab*):ti,ab,kw  4 (severe stroke impair*):ti,ab,kw  5 (severe stroke limit*):ti,ab,kw  6 #1 OR #2 OR #3 OR #4 OR #5  7 MeSH descriptor: [Autogenic Training] explode all trees  8 MeSH descriptor: [Combined Modality Therapy] explode all trees  9 MeSH descriptor: [Exercise Movement Techniques] explode all trees  10 MeSH descriptor: [Mentoring] explode all trees  11 MeSH descriptor: [Nursing Care] explode all trees  12 MeSH descriptor: [Patient Positioning] explode all trees  13 MeSH descriptor: [Stroke Rehabilitation] explode all trees  14 MeSH descriptor: [Teaching] explode all trees  15 MeSH descriptor: [Transcutaneous Electric Nerve Stimulation] explode all trees | 16 MeSH descriptor: [Video Games] explode all trees  17 MeSH descriptor: [Virtual Reality Exposure Therapy] explode all trees  18 (aerobic exercise):ti,ab,kw  19 (aerobic training):ti,ab,kw  20 (biofeedback):ti,ab,kw  21 (coaching):ti,ab,kw  22 (cognitive behavioral therapy):ti,ab,kw  23 (cognitive rehabilitation):ti,ab,kw  24 (constraint induced therapy):ti,ab,kw  25 (education):ti,ab,kw  26 (electric stimulation therapy):ti,ab,kw  27 (exercise):ti,ab,kw  28 (exercise therapy):ti,ab,kw  29 (functional electric stimulation):ti,ab,kw  30 (health education):ti,ab,kw | 31 (home care):ti,ab,kw  32 (home rehabilitation):ti,ab,kw  33 (intensive care):ti,ab,kw  34 (mirror therapy):ti,ab,kw  35 (mobilisation):ti,ab,kw  36 (motor relearning program):ti,ab,kw  37 (movement therapy):ti,ab,kw  38 (muscle training):ti,ab,kw  39 (neuromuscular electric stimulation):ti,ab,kw  40 (nursing):ti,ab,kw  41 (occupational therapy):ti,ab,kw  42 (patient education):ti,ab,kw  43 (physical activity):ti,ab,kw  44 (physical therapy modalities):ti,ab,kw  45 (physical therapy speciality):ti,ab,kw | 46 (robotics):ti,ab,kw  47 (task specific training):ti,ab,kw  48 (virtual reality):ti,ab,kw  49 #7 OR #8 OR #9 OR #10 OR #11 OR #12 OR #13 OR #14 OR #15 OR #16 OR #17 OR #18  OR #19 OR #20 OR #21 OR #22 OR #23 OR #24 OR #25 OR #26 OR #27 OR #28 OR #29  OR #30 OR #31 OR #32 OR #33 OR #34 OR #35 OR #36 OR #37 OR #38 OR #39 OR #40  OR #41 OR #42 OR #43 OR #44 OR #45 OR #46 OR #47 OR #48  50 (randomized controlled trial):ti,ab,kw  51 (RCT):ti,ab,kw  52 #50 OR #51  53 #6 AND #49 AND #52  54 #6 AND #49 AND #52, Filter: in Trials  55 #6 AND #49 AND #52, Filter: in Trials, 2018--2024 |
| *International Clinical Trials Registry Platform* | | | |
| 1 severe stroke (Title)  Recruitment status: ALL  2 stroke severity (Title)  Recruitment status: ALL  3 severe stroke impair* (Title)  Recruitment status: ALL  4 stroke disab* (Title)  Recruitment status: ALL  5 #1 OR #2 OR #3 OR #4  Recruitment status: ALL  6 #1 OR #2 OR #3 OR #4  Recruitment status: ALL  Filter: Date of Registration 01/01/2018 bis 06/09/2024 | | | |
